# The “backdoor pathway” of androgen synthesis in human male sexual development

**DOI:** 10.1371/journal.pbio.3000198

**Published:** 2019-04-03

**Authors:** Walter L. Miller, Richard J. Auchus

**Affiliations:** 1 Department of Pediatrics and Center for Reproductive Sciences, University of California-San Francisco, San Francisco, California, United States of America; 2 Departments of Medicine and of Pharmacology, University of Michigan, Ann Arbor, Michigan, United States of America

## Abstract

Mammalian sex determination (male versus female) is largely controlled by genes, whereas sex differentiation (development of reproductive structures) is largely controlled by hormones. Work in the 20th century indicated that female external anatomy was a “default” pathway of development not requiring steroids, whereas male genital development required testicular testosterone plus dihydrotestosterone (DHT) made in genital skin according to a “classic” pathway. Recent work added the description of an alternative “backdoor” pathway of androgen synthesis discovered in marsupials. Unique “backdoor steroids” are found in human hyperandrogenic disorders, and genetic disruption of the pathway causes disordered male sexual development, suggesting it plays an essential role. O’Shaughnessy and colleagues now show that the principal human backdoor androgen is androsterone and provide strong evidence that it derives from placental progesterone that is metabolized to androsterone in nontesticular tissues. These studies are essential to understanding human sexual development and its disorders.

## Abstract 2

Female external anatomy is the ‘default’ pathway of development, while male genital development requires testicular testosterone plus dihydrotestosterone made in genital skin. This Primer explores recent evidence that an additional 'backdoor' pathway to masculinization involves androsterone, derived from placental progesterone.

Sexual selection and the importance of sexually dimorphic displays and reproductive structures were first considered by Darwin and Wallace [1]. We now know that these phenotypes are largely determined by sex steroids; several sex steroids (estrone, androsterone, progesterone) were isolated in the 1920s by Leopold Ruzika and Alfred Butenandt, who shared the 1939 Nobel Prize for their discovery. The founder of statistical genetics, Roland Fisher, combined Darwinian natural selection with Mendelian genetics and proposed that these newly discovered sex hormones acted separately from genes, permitting the distinction between sexual determination (from genes on sex chromosomes) and sexual differentiation (induced by hormones) [2]. This incisive synthesis remained unproven until Alfred Jost showed that testicular androgens are required for the differentiation of embryonic Wolffian ducts into male reproductive structures and proposed that the testis also produced a locally acting “Müllerian inhibitory substance” (now called “anti-Müllerian hormone” [AMH]) that was responsible for the involution of the structures that would otherwise differentiate into a uterus and Fallopian tubes [3,4]. Two more recent events seem to cement this model. First, after much effort, AMH was isolated, cloned, and shown to act precisely as Jost had proposed [5,6]. Second, Siiteri and Wilson discovered that testosterone secreted by the testis could be 5α-reduced to a more potent androgen, dihydrotestosterone (DHT), in genital skin [7], leading to the “two-androgen” model of male sexual differentiation, in which testosterone virilizes the Wolffian ducts (to form the seminal vesicle and ejaculatory ducts) and DHT virilizes the male external genitalia and urethra [8]. But this model was not strictly proven and required examination of the hormones produced in the testis and genital skin during early fetal development, and for this purpose, conventional laboratory animals proved cumbersome at best.

## The “classic” and “backdoor” pathways of androgen synthesis

**Fig 1:**
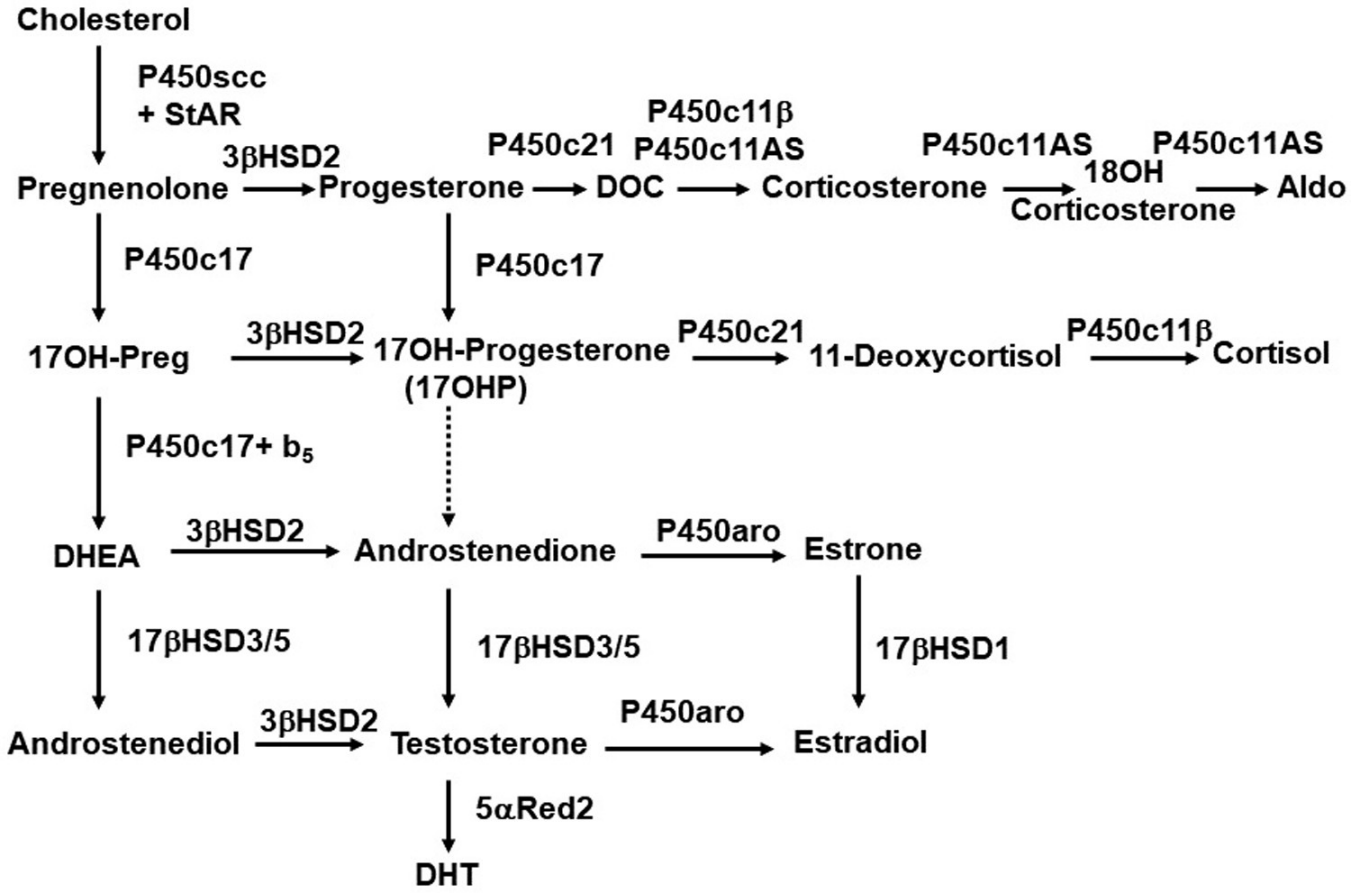
The “conventional” pathway of steroidogenesis. The figure combines adrenal and gonadal pathways. The left-hand column shows the ∆5 pathway, in which steroids retain the double bond between carbons 5 and 6 in cholesterol’s B-ring. The StAR protein facilitates import of cholesterol into mitochondrial, in which P450scc (CYP11A1) cleaves off the side-chain to yield Preg, the first C21 steroid. ∆5 steroids are converted to corresponding ∆4 steroids by 3βHSD2 (HSD3B2) in the adrenal and gonad or by 3βHSD1 in placenta and peripheral tissues. In the absence of P450c17 (in the adrenal zona glomerulosa), progesterone is 21-hydroxylated by P450c21 (CYP21A2). P450c11AS (Aldo synthase, CYP11B2) then catalyzes 11-hydroxylase, 18-hydroxylase, and 18-methyl oxidase activities to yield Aldo. In the gonads and adrenal zona fasciculata, the 17α-hydroxylase activity of P450c17 (CYP17A1) permits synthesis of 17OHP, which is 11-hydroxylated to cortisol by P450c11β (CYP11B1). The 17,20 lyase activity of P450c17 requires allosteric action of cytochrome b5 (b5) in the adrenal zona reticularis and testicular Leydig cells, permitting conversion of C21 to C19 steroids. Human P450c17 converts 17OHP to androstenedione with only approximately 2–3% of its activity to convert 17OH-Preg to DHEA so that testosterone synthesis proceeds via DHEA and not via 17OHP; by contrast, rodent and ungulate P450c17 catalyzes this reaction efficiently. Testicular 17βHSD3 converts DHEA to androstenediol and androstenedione to testosterone; low levels of adrenal 17βHSD5 (AKR1C3) permit synthesis of small amounts of testosterone. In the ovary and elsewhere, P450aro (aromatase, CYP19A1) converts C19 androgens to C18 estrogens. In genital skin, 5α-reductase type 2 (5αRed2, SRD5A2) further activates testosterone to DHT. 17OHP, 17OH-progesterone; Aldo, aldosterone; C21, 21-carbon; DHEA, dehydroepiandrosterone; DHT, dihydrotestosterone; Preg, pregnenolone; StAR, steroidogenic acute regulatory protein.

Clinical studies, studies of human tissues, and studies of conventional laboratory animals had converged to a classical model of androgen synthesis, both in the testis and in a zone of the primate adrenal cortex called the zona reticularis [9]. Cholesterol is first converted to pregnenolone (Preg) by the cholesterol side-chain cleavage enzyme P450scc (CYP11A1); the 17α-hydroxylase activity of P450c17 (CYP17A1) then converts Preg to 17OH-Preg, then the 17,20 lyase activity of P450c17 converts 17OH-Preg to dehydroepiandrosterone (DHEA). DHEA is converted to androstenedione by 3β-hydroxysteroid dehydrogenase type 2 (HSD3B2) in both the adrenal and testis, but the testis is the major site of the next reaction: conversion of androstenedione to testosterone by 17β-hydroxysteroid dehydrogenase type 3 (HSD17B3). The resulting testosterone is secreted into the circulation and taken up by genital skin, in which it is converted to DHT by 5α-reductase type 2 (SRD5A2) (Fig 1).

**Fig 2:**
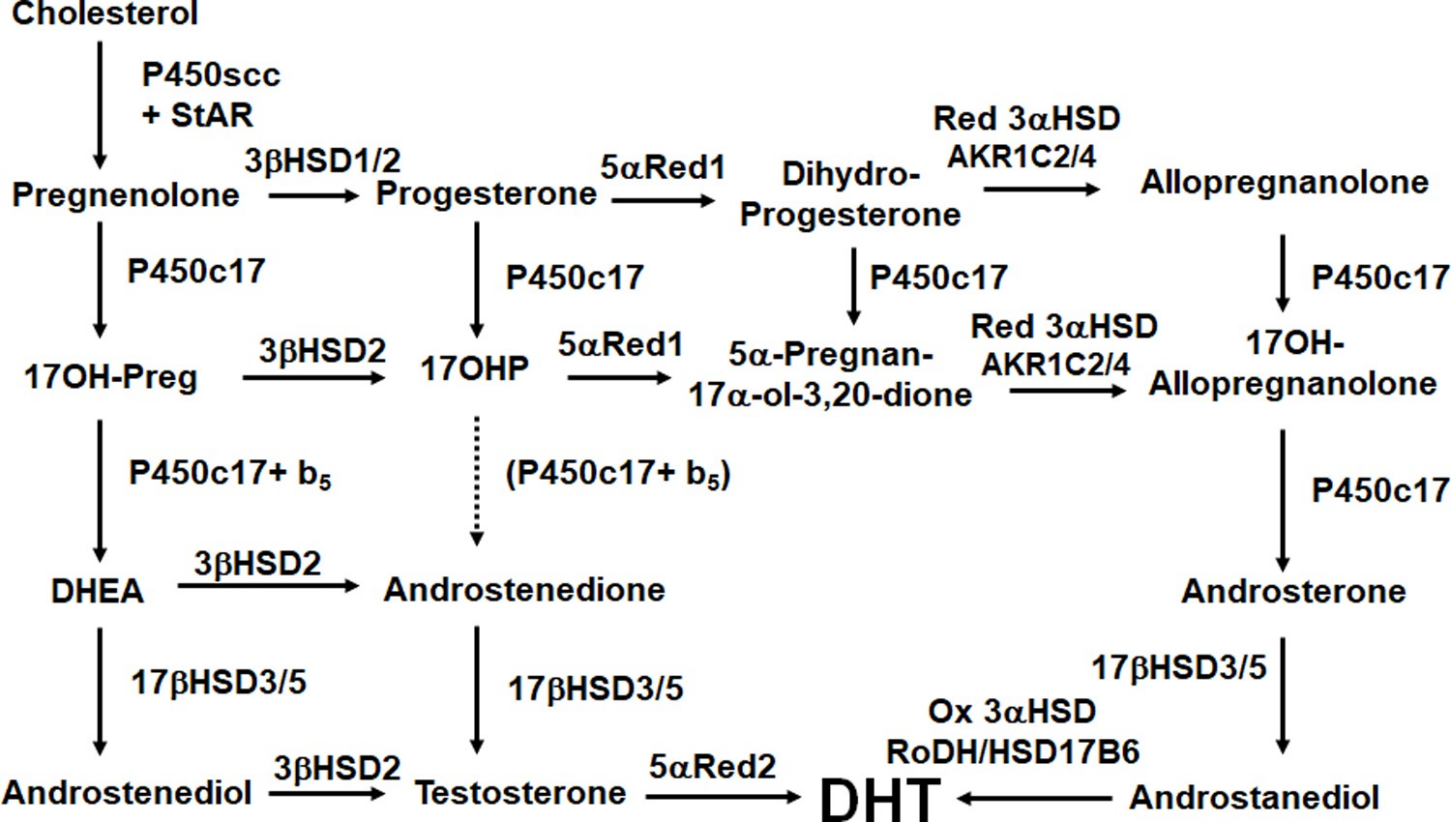
The “backdoor” pathway of androgen synthesis. Steroids in the left-hand column (the Δ5 pathway) may be acted on by either 3βHSD1 or 3βHSD2 to yield the corresponding Δ4 steroids. Following the production of Preg, the backdoor pathway typically features its conversion to 17OH-Preg, which is then converted to the key intermediary, 17OHP. In the brain (and elsewhere), progesterone may be converted to the neuroactive steroid, allopregnanolone. 17OHP is 5α-reduced by 5αRed1 (SRD5A1) to 5α-pregnan-17α-ol-3,20-dione, which is then 3α-reduced by AKR1C2 or AKR1C4 to yield 17OH-allopregnanolone. P450c17 catalyzes its 17,20 lyase activity very efficiently when 17OH-allopregnanolone is the substrate, yielding androsterone, which O’Shaughnessy and colleageus show is the principal androgen in human male fetal circulation. Androsterone may then acted on by testicular 17βHSD3 (or, to a minor degree, by adrenal 17βHSD5 [AKR1C3]) to yield androstanediol, which may be 3α-oxidized, probably by 17βHSD6 (HSD17B6; also known as RoDH, to yield the most potent androgen, DHT. The work of O’Shaughnessy and colleagues shows that the human fetal testis instead uses progesterone produced by the placenta to generate the 17OHP that initiates the backdoor pathway. The identities of all of the enzymes catalyzing the reductive and oxidative 3αHSD reactions have not been determined unambiguously. 17OHP, 17OH-progesterone; DHT, dihydrotestosterone; Preg, pregnenolone; RoDH, retinol dehydrogenase.

The pioneering studies of Marilyn Renfree provided an animal model in which the accuracy of this pathway in the developing male could be tested: the Tammar wallaby [10–12]. Marsupial mammals such as the wallaby differ from more familiar eutherian (placental) mammals in that gonadal differentiation and sexual development take place after birth, while the newborn is in the mother’s pouch, where the young are readily accessible for tissue sampling and where drugs and hormones may be administered without placental intervention. Thus, the wallaby provided the means for testing the “conventional wisdom” that testosterone is the androgen in the circulation while DHT is made and acts locally in the genital skin. The wallaby did not cooperate! Working with Renfree, Wilson, and colleagues discovered that the wallaby produces DHT by a steroidogenic pathway that had not been seen in eutherian mammals. This pathway bypasses the usual intermediate steroids DHEA, androstenedione and testosterone. Instead, 17OH-progesterone is made from 17OH-Preg, then 5α- and 3α- are reduced, subjected to the 17,20 lyase activity of P450c17, then acted on by HSD17B3 to produce 5α-androstane-3α,17β-diol as the circulating product. Similar to the eutherian mammal, the target tissues perform the final enzymatic transformation: 3α-oxidation to produce DHT [13]. This novel alternate pathway to DHT thus circumvents the usual steroidal intermediates and has come to be known as “the backdoor pathway” [14] (Fig 2).

## Role of the “backdoor” pathway in human male development

Indirect data supporting the relevance of the backdoor pathway in human physiology are emerging. Studies measuring steroidal intermediates of the backdoor pathway by liquid chromatography coupled with tandem mass spectrometry have indicated that this pathway plays an important role in human hyperandrogenic disorders such as congenital adrenal hyperplasia caused by steroid 21-hydroxylase deficiency [15], the polycystic ovary syndrome [16], and some virilized female newborns with P450 oxidoreductase deficiency [17–19], as well as in the physiologic male “minipuberty of infancy” [20]. Furthermore, mutations causing disordered sexual development have been found in the *AKR1C2* and *AKR1C4* genes that encode the 3α-reductases that function uniquely in this pathway [21], leading to the conclusion that both the “classic” pathway of androgen synthesis and the “backdoor” pathway are needed for normal human male genital development [22]. Nevertheless, these studies provided only indirect evidence, as androgens in the circulation and tissues were not measured in these fetuses.

## The major human “backdoor” androgen is androsterone, derived from placental progesterone

In the current issue of *PLOS Biology*, O’Shaughnessy and colleagues addressed the problem directly by measuring the plasma and tissue concentrations of steroids from the conventional and backdoor pathways in 42 midgestation human male fetuses [23]. They found that the principal circulating androgen is androsterone rather than testosterone and that DHT was below the limit of detection in blood. As expected, concentrations of androsterone and testosterone were lower in blood from female fetuses. Rather surprisingly, levels of androsterone and steroidal intermediates specific to the backdoor pathway were found mainly in the placenta, liver, and adrenal rather than the testis; furthermore, PCR analyses showed similar distributions of the mRNAs for P450c17, SRD5A2, and AKR1C2/4 in these tissues, suggesting that these tissues were the sites where these steroids were synthesized. Thus, the data are consistent with an essential role for the backdoor pathway in producing the androgens that virilize the male fetus but indicate that this androgenesis is distributed among multiple tissues, starting with placentally produced progesterone rather than being confined to testicular Leydig cells, as has been traditionally envisioned.

Removing the testis from the center of fetal androgen synthesis is a novel, unexpected finding. The work of O’Shaughnessy and colleagues confirms the “two-androgen” model of male sexual development wherein testosterone is sufficient to stabilize and differentiate the Wolffian ducts and to promote penile growth, while DHT is needed to virilize the external genitalia. But the testis is no longer supreme: it is necessary but not sufficient to produce the androgenic steroids that are responsible for male sexual development.

The study was limited to a modest number of fetuses at estimated gestational ages of 11–21 weeks, and concentrations of many of the analytes were near the lower limits of assay detection. Nevertheless, the specimens used by the authors are difficult to obtain, and the authors carefully attempted to control for many variables, including fetal sex. Future studies will need to address the flux of steroids in the various tissues along various steroidogenic steps. The unexpected results of this study will inspire additional work to unravel the proportions of androgens derived from the traditional and alternative pathways across gestation and their relative contributions to male sexual development in the normal fetus and in disorders of sex development.

## Abbreviations

AMH: anti-Müllerian hormone
CYP11A1: cholesterol side-chain cleavage enzyme P450scc
CYP17A1: 17α-hydroxylase/17,20 lyase (P450c17)
DHEA: dehydroepiandrosterone
DHT: dihydrotestosterone
HSD3B2: 3β-hydroxysteroid dehydrogenase type 2
HSD17B3: 17β-hydroxysteroid dehydrogenase type 3
Preg: pregnenolone
SRD5A2: 5α-reductase type 2
